# Effects of total temporomandibular joint replacement with alloplastic prosthesis – a systematic review

**DOI:** 10.2340/aos.v84.43641

**Published:** 2025-05-20

**Authors:** Pär Morin, Jani Talvilahti, Mattias Ulmner, Per Alstergren, Eva Nordendahl, Aron Naimi-Akbar

**Affiliations:** a Health Technology Assessment-Odontology (HTA-O), Faculty of Odontology, Malmö University, Malmö, Sweden; b Region Dalarna, Department of Oral and Maxillofacial Surgery, Falun, Sweden; c Medical Unit for Reconstructive Plastic- and Craniofacial Surgery, Karolinska University Hospital, Stockholm, Sweden; d Division of Oral Diagnostics and Rehabilitation, Department of Dental Medicine, Karolinska Institute, Stockholm, Sweden; e Faculty of Odontology, Malmö University, Malmö, Sweden; f Department of Periodontology, Eastman Institute, Folktandvården Stockholm AB, Stockholm, Sweden

**Keywords:** Total joint replacement, patient reported outcome measures, quality of life, temporomandibular joint, temporomandibular joint disorders, arthroplasty, replacement

## Abstract

**Objectives:**

This paper studies the treatment effect of total joint replacement (TJR) of the temporomandibular joint (TMJ) with alloplastic joint prosthesis regarding function, symptoms and health-related quality of life compared to other surgical and non-surgical treatments in patients with TMJ disorders.

**Methods:**

Three databases (PubMed, Cochrane Library and Web of Science) were searched up to 11 March 2025. Studies in which TJR was compared with other surgical or non-surgical methods were searched and analyzed. Data extraction and quality assessments were performed by at least two investigators independently. Risk of bias was assessed with the ROBINS-I-tool. Certainty of evidence was assessed with GRADE.

**Results:**

A total of 2,891 studies were identified in the search. One study met the criteria with comparison of TJR with a control group consisting of patients treated with another surgical method, namely, interpositional arthroplasty. The study investigated the outcome variables such as pain reduction and improvement in mouth opening and had a moderate risk of bias. No significant difference between the groups was found after regression analysis. Quality of life assessment was not the objective of this study. No meta-analysis could be performed from this literature search, for obvious reasons.

**Conclusion:**

TMJ reconstruction with alloplastic prostheses is rapidly evolving, with new systems continually entering the market. This review highlights the urgent need for further scientific efforts, including well-designed trials capable of demonstrating the comparative effectiveness of alloplastic TJR against other treatment modalities, ideally randomized trials with controls.

## Introduction

The temporomandibular joint (TMJ) is a functional complex joint that serves to facilitate movement of the jaw and thereby allows speech, chewing etc. The TMJ is subject to local or systemic diseases and disorders as other joints in the human body. Temporomandibular disorders (TMDs) is an umbrella term for musculoskeletal disorders in the orofacial region causing pain and dysfunction [1]. TMDs are common conditions, affecting 30% of the adult population [2, 3] with the highest incidence from adolescence to 60 years of age, and affect women to a higher extent [4]. These conditions can eventually lead to cartilage and bone tissue destruction, adhesions and/or heterotrophic bone formation [5]. Dysfunction of the TMJ, for example internal derangements, is associated with pain and restrictions of the jaws [6]. There are reasons to believe that dysfunction of the TMJ also leads to poor health-related quality of life (QoL) due to the many situations where jaw function is crucial.

Most patients with TMJ disease will benefit from conservative treatment, arthrocentesis [7], or arthroscopy [8, 9]; but in more severe conditions with, for example, severe TMJ destruction or ankylosis, more invasive surgical treatments are indicated [10–12]. The surgical treatments aim to re-establish anatomy and function, which may in turn reduce pain in end-stage conditions of the TMJ. For the most severe conditions, there might be a need for total joint replacement (TJR) of the TMJ with alloplastic total joint prosthesis [13].

TJR of the TMJ with alloplastic joint prosthesis is a surgically advanced treatment for managing severe disease or deformity or dysfunction of the TMJ [14]. The TJR procedure implies replacement of the condylar head and temporal fossa with alloplastic material [15]. Both alloplastic materials as well as autologous materials and techniques have been proposed and used for arthroplastic reconstructive surgery for complex conditions of the TMJ. Gap osteotomies and autologous methods such as costochondral grafts [16] and temporalis muscle flap have been, and are still, used [17]. Different types of alloplastic prostheses have been used for severe conditions of the TMJ [18]. There has been a number of devices with the purpose of replacing the condyle alone and as TJR with both condyle and fossa replacement [19]. Today, the most commonly used joint prosthesis of the TMJ is a device with a mandibular component made of cobalt-chrome molybden/titanium and a fossa component of ultra-high-molecular-weight polyethylene. The TJR with alloplastic joint prosthesis has shown promising results, and there are indications that it could be considered reliable over time [13].

One desired goal of the alloplastic device is to re-establish the anatomy and function and to prevent malocclusion from the lack of posterior height in the ramus [20]. Using such an alloplastic device for reconstruction of the TMJ might decrease the risk of further tissue degeneration or other local pathology and pain, which has been shown for autologous grafts and arthroplasty [21, 22].

**Table 1:** Eligibility criteria.

Criteria	Description
Research question	What is the treatment effect of total joint replacement with alloplastic joint prosthesis regarding function, symptoms and quality of life compared to other surgical and non-surgical treatments in patients with temporomandibular joint disorders?
Population	Patients with any type of temporomandibular joint disorders, eg. acquired deformities or disease of the temporomandibular joint, congenital deformities or disease of the temporomandibular joint.
Intervention	Total joint replacement with alloplastic joint prosthesis of the temporomandibular joint.
Control	Physiotherapy including acupuncture etc. Plastic or reconstructive surgery of the temporomandibular joint Local injection therapy Other surgical interventionof temporomandibular joint or tissues surrounding temporomandibular joint. Different types of total joint prosthesis Dental appliances/splint No treatment
Outcome	Health-related quality of life or other patient-related outcome measures
	Measures of function
	Measures of symptoms or pain
	Other measures of temporomandibular joint disease or dysfunction
Inclusion criteria	Clinical longitudinal studies with a control group such as randomized controlled trial, controlled clinical trial or cohort studies.
	Systematic reviews concerning the same field of interest
Exclusion criteria	Publications in languages other than English or Scandinavian languages.
	Publications with treatment without controls

To our knowledge, there is no systematic review (SR) that examines the effectiveness of TJR with alloplastic joint pro-sthesis to treat severe TMJ diseases with QoL as the primary outcome measure. Therefore, the aim of this paper is to analyze the scientific evidence for this treatment in a SR.

## Methods

This paper was written in accordance with Preferred Reporting Items for Systematic Reviews and Meta-Analysis (PRISMA) checklist. The study protocol was registered on the international prospective register of systematic reviews (PROSPERO) (https://www.crd.york.ac.uk/prospero/) with the protocol no. CRD42020195987.

### Research question

What is the treatment effect of TJR with alloplastic joint prosthesis regarding function, symptoms, and QoL, compared to other surgical and non-surgical treatments in patients with severe TMJ dysfunction?

### Eligibility criteria

PICO is a framework for structured research questions by outlining the Population (P), Intervention (I), Comparison (C), and Outcome (O) of interest.

#### Population

Patients with severe disorders, acquired deformities or disease of the TMJ, congenital deformities or disease of the TMJ.

#### Intervention

TJR with alloplastic joint prosthesis of the TMJ.

#### Control

Controls are patients receiving one or more of the following approaches for managing TMJ disorders:

Plastic or reconstructive surgery of the TMJ

Physiotherapy interventions (including acupuncture)

Injection treatments

Other surgical procedures targeting the TMJ or surrounding tissues

No active treatment (observation only)

#### Outcomes

QoL or other patient-related outcome measures. Measures of TMJ function, symptoms or pain, other measures of TMJ disease or dysfunction.

We included clinical longitudinal studies with a control group such as randomized controlled trials, controlled clinical trials or cohort studies.

## Literature search

The literature search was performed in PubMed, Cochrane Library and Web of Science. The reference lists of the included papers were hand-searched for relevant literature. Only studies in English and Scandinavian languages were included. Details of the search strategy for the different databases are presented in Table 2.

**Table 2:** Literature search strategy.

**PubMed via NLM** 250311		
1	TMD[Title/Abstract] OR TMJD[Title/Abstract] OR TMJ disorder*[Title/Abstract] OR TMJ disease*[Title/Abstract] OR TMJ syndrome*[Title/Abstract] OR TMJ dysfunction*[Title/Abstract] OR temporomandibular joint disorder*[Title/Abstract] OR temporomandibular disorder*[Title/Abstract] OR temporomandibular joint disease*[Title/Abstract] OR temporomandibular joint dysfunction syndrome*[Title/Abstract] OR temporomandibular joint syndrome*[Title/Abstract] OR temporomandibular joint dysfunction*[Title/Abstract] OR temporomandibular dysfunction*[Title/Abstract] OR Craniomandibular Disorder*[Title/Abstract] OR Craniomandibular Dysfunction*[Title/Abstract] OR temporomandibular joint disorders[MeSH Terms] OR Craniomandibular Disorders[MeSH Terms] OR ((osteoarthritis[Title/Abstract] OR osteoarthritis[MeSH Terms] ankylosis[Title/Abstract] OR ankylosis[MeSH Terms] OR “condylar resorption”[Title/Abstract] OR “condylar hypoplasia”[Title/Abstract] OR arthritis[Title/Abstract] OR arthritis[MeSH Terms]) AND (temporomandibular[Title/Abstract]))	29 113
2	Arthroplasty, Replacement[MeSH Terms] OR joint replacement*[Title/Abstract] OR joint prosthes*[Title/Abstract] OR joint reconstruction*[Title/Abstract] OR joint plast*[Title/Abstract] OR alloplastic joint*[Title/Abstract] OR “Temporomandibular Joint/Surgery”[MeSH Terms]	88 570
3	1 AND 2	1 699
**Cochrane via Wiley** 250311		
1	ti,ab,kw(TMD OR TMJD OR TMJ disorder* OR TMJ disease* OR TMJ syndrome* OR TMJ dysfunction* OR temporomandibular joint disorder* OR temporomandibular disorder* OR temporomandibular joint disease* OR temporomandibular joint dysfunction syndrome* OR temporomandibular joint syndrome* OR temporomandibular joint dysfunction* OR temporomandibular dysfunction* OR Craniomandibular Disorder* OR Craniomandibular Dysfunction*) OR MeSH descriptor: [Craniomandibular Disorders] OR [Temporomandibular Joint Disorders] explode all trees	2 735
2	(Ti,ab,kw(osteoarthritis OR ankylosis OR condylar resorption OR condylar hypoplasia OR arthritis) OR MeSH descriptor: [Arthritis] OR [Ankylosis] OR [Osteoarthritis] explode all trees) AND (temporomandibular):ti,ab,kw	240
3	1 OR 2	2 774
4	ti,ab,kw (joint replacement* OR joint prosthes* OR joint reconstruction* OR joint plast* OR alloplastic joint*) OR MeSH descriptor: [Arthroplasty] OR [Temporomandibular Joint] explode all trees and with qualifier(s): [surgery – SU]	13 080
5	3 AND 4	145
	Cochrane reviews – 2, trials 143	
**Web of Science via Clarivate** 250311		
1	TMD OR TMJD OR TMJ disorder* OR TMJ disease* OR TMJ syndrome* OR TMJ dysfunction* OR temporomandibular joint disorder* OR temporomandibular disorder* OR temporomandibular joint disease* OR temporomandibular joint dysfunction syndrome* OR temporomandibular joint syndrome* OR temporomandibular joint dysfunction* OR temporomandibular dysfunction* OR Craniomandibular Disorder* OR Craniomandibular Dysfunction* OR ((osteoarthritis OR ankylosis OR condylar resorption OR condylar hypoplasia OR arthritis) AND (temporomandibular)) (topic)	29 889
2	joint replacement* OR joint prosthes* OR joint reconstruction* OR joint plast* OR alloplastic joint* (topic)	92 103
3	1 AND 2	1 357

TMD: Temporomandibular disorders.

### Selection

The identified publications from the literature search were subjected to an initial screening process based on title and abstract using the Rayyan software (https://www.rayyan.ai/). The titles and abstracts were read independently by at least two reviewers. Selected publications were retrieved and read in full-text by each reviewer independently and each publication by at least two reviewers. Any potential disagreement during any stage of the screening process was resolved through a consensus discussion between the authors. The list of excluded studies after being read in full-text is presented in Table 3.

**Table 3:** Studies excluded after read in full text (n = 19) and reason for exclusion.

Study	Reason for exclusion
Alakailly X, Schwartz D, Alwanni N, Demko C, Altay MA, Kilinc Y, et al. Patient-centered quality of life measures after alloplastic temporomandibular joint replacement surgery. Int J Oral Maxillofac Surg. 2017;46(2):204–7.	Wrong control
Alam MK, Rashid ME, Akhter K, Abdelghani A, Babkair HA, Sghaireen MG. Surgical vs. Non-Surgical Management of Temporomandibular Joint Disorders: Clinical Outcomes. J Pharm Bioallied Sci. 2024;16:S678–s680.	Wrong intervention
Elledge R, Attard A, Green J, Lowe D, Rogers SN, Sidebottom AJ, et al. UK temporomandibular joint replacement database: a report on one-year outcomes. Br J Oral Maxillofac Surg. 2017;55(9):927–31.	Wrong control
Gerbino G, Zavattero E, Bosco G, Berrone S, Ramieri G. Temporomandibular joint reconstruction with stock and custom-made devices: indications and results of a 14-year experience. J Craniomaxillofac Surg. 2017;45(10):1710–5.	Wrong control
Gundlach KK. Ankylosis of the temporomandibular joint. J Craniomaxillofac Surg. 2010;38(2):122–30.	Wrong type of study
Handa S, Youness M, Keith DA, Rosén A. Persistent pain after total temporomandibular joint replacement surgery: clinical characteristics, comorbidities, and risk factors. Int J Oral Maxillofac Surg. 2025;54(2):166–73.	Wrong control
House LR, Morgan DH, Hall WP, Vamvas SJ. Temporomandibular joint surgery: results of a 14-year joint implant study. Laryngoscope. 1984;94(4):534–8.	Wrong outcome
Idle MR, Lowe D, Rogers SN, Sidebottom AJ, Speculand B, Worrall SF. UK temporomandibular joint replacement database: report on baseline data. Br J Oral Maxillofac Surg. 2014;52(3):203–7.	Wrong control
Jones RH. Temporomandibular joint reconstruction with total alloplastic joint replacement. Aust Dent J. 2011;56(1):85–91.	Wrong control
Kummoona R. Temporomandibular joint reconstruction with a 2-part chrome-cobalt prosthesis, chondro-osseous graft, and silastic: clinical and experimental studies. J Craniofac Surg. 2009;20(6):2125–35.	Wrong control
*Mansuri S, Hemavathy S, Tejaswee ASS, et al. Comparison of Surgical Techniques for Correction of Mandibular Asymmetry in TMJ Ankylosis Patients. JOURNAL OF PHARMACY AND BIOALLIED SCIENCES. 2024;16:S2363–5.*	Wrong outcome
McKenzie WS, Louis PJ. Temporomandibular total joint prosthesis infections: a ten-year retrospective analysis. Int J Oral Maxillofac Surg. 2017;46(5):596–602.	Wrong outcome
Posnick JC, Jacobs JS, Magee WP, Jr. Prosthetic replacement of the condylar head for temporomandibular joint disease. Plast Reconstr Surg. 1987;80(4):536–46.	Wrong intervention
Schiffman EL, Look JO, Hodges JS, Swift JQ, Decker KL, Hathaway KM, et al. Randomized effectiveness study of four therapeutic strategies for TMJ closed lock. J Dent Res. 2007;86(1):58–63.	Wrong intervention
Schiffman EL, Velly AM, Look JO, Hodges JS, Swift JQ, Decker KL, et al. Effects of four treatment strategies for temporomandibular joint closed lock. Int J Oral Maxillofac Surg. 2014;43(2):217–26.	Wrong intervention
Speculand B, Hensher R, Powell D. Total prosthetic replacement of the TMJ: experience with two systems 1988–1997. Br J Oral Maxillofac Surg. 2000;38(4):360–9.	Wrong control
Valentini V, Vetrano S, Agrillo A, Torroni A, Fabiani F, Iannetti G. Surgical treatment of TMJ ankylosis: our experience (60 cases). J Craniofac Surg. 2002;13(1):59–67.	Wrong intervention
Vasconcelos BC, Porto GG, Bessa-Nogueira RV. Temporo mandibular joint ankylosis. Braz J Otorhinolaryngol. 2008;74(1):34–8.	Wrong intervention
Zhang W, Yang X, Zhang Y, Zhao T, Jia J, Chang S, et al. The sequential treatment of temporomandibular joint ankylosis with secondary deformities by distraction osteogenesis and arthroplasty or TMJ reconstruction. Int J Oral Maxillofac Surg. 2018;47(8):1052–9.	Wrong intervention

### Data extraction

The relevant data in the included studies were extracted by one of the authors (PM), and the data extraction was checked by another author (ANA). Information about author names, publication year, country, study design, study population (age, sex, general health, TMJ-diseases), type of intervention and control, number of included patients and follow-up time, outcome measures, effect measures and relevant results was extracted.

### Risk of bias assessment

The risk of bias in the included non-randomized studies was assessed using the ROBINS-I tool [23]. Before the assessment, the following confounding factors were specified as important: severity of TMJ disorder, number of preceding surgical procedures, pain prior to intervention, age, general health, smoking, and gender. At least two of the authors independently evaluated the studies and assessed the risk of bias of the included studies. If the risk of bias was determined as critical, the study was excluded.

### Synthesis methods

Only one study was included in the final analysis, thus no synthesis was made.

### Reporting bias assessment

Only one study was included, thus no reporting bias assessment was made.

### Certainty assessment

The certainty of evidence was assessed according to GRADE [24] as high, moderate, low or very low.

## Results

### Study selection

**Figure 1:**
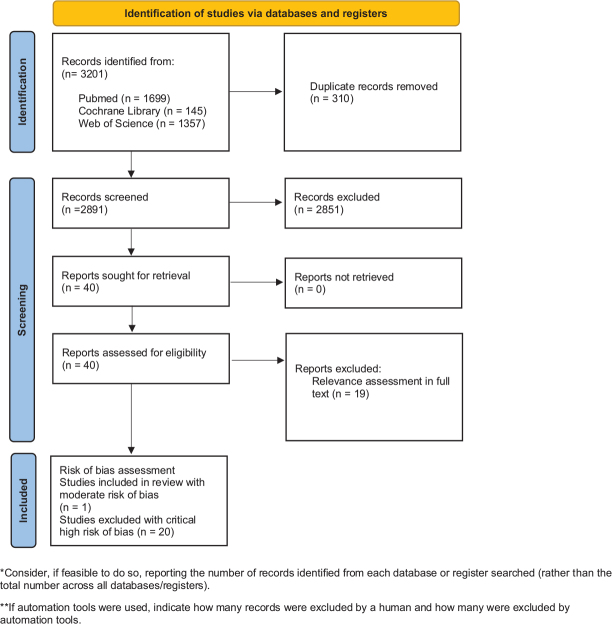
PRISMA 2020 flow diagram.

The result of the selection process is presented in Figure 1. A total of 2,891 studies were identified after removing 310 duplicates. After reading all titles and abstracts, 40 studies were included to be read in full text. Nineteen studies were excluded after full-text eligibility assessment, and the reasons for exclusion can be viewed in Table 3. Twenty-one studies were assessed for risk of bias and only one study was found to meet the criteria for moderate risk of bias (Table 4).

**Table 4:** Studies with unacceptable high risk of bias (n = 20).

Study	Domain with critical high risk of bias
Burgess M, Bowler M, Jones R, Hase M, Murdoch B. Improved outcomes after alloplastic TMJ replacement: analysis of a multicenter study from Australia and New Zealand. J Oral Maxillofac Surg. 2014;72(7):1251–7.	Confounding
Chase DC, Hudson JW, Gerard DA, Russell R, Chambers K, Curry JR, et al. The Christensen prosthesis. A retrospective clinical study. Oral Surg Oral Med Oral Pathol Oral Radiol Endod. 1995;80(3):273–8.	Confounding
Chen S, He Y, An JG, Zhang Y. Recurrence-related factors of temporomandibular joint ankylosis: a 10-year experience. J Oral Maxillofac Surg. 2019;77(12):2512–21.	Confounding
Dimitroulis G. Comparison of the outcomes of three surgical treatments for end-stage temporomandibular joint disease. Int J Oral Maxillofac Surg. 2014;43(8):980–9.	Confounding
Gerbino G, Zavattero E, Berrone S, Ramieri G. One stage treatment of temporomandibular joint complete bony ankylosis using total joint replacement. J Craniomaxillofac Surg. 2016;44(4):487–92.	Selection
Gerbino G, Zavattero E, Berrone S, Ramieri G. One stage treatment of temporomandibular joint complete bony ankylosis using total joint replacement. J Craniomaxillofac Surg. 2016;44(4):487–92.	Confounding
Gonzalez-Perez LM, Gonzalez-Perez-Somarriba B, Centeno G, Vallellano C, Montes-Carmona JF. Evaluation of total alloplastic temporo-mandibular joint replacement with two different types of prostheses: a three-year prospective study. Med Oral Patol Oral Cir Bucal. 2016;21(6):e766–e75.	Confounding
Gonzalez-Perez LM, Gonzalez-Perez-Somarriba B, Centeno G, Vallellano C, Montes-Carmona JF, Torres-Carranza E, et al. Prospective study of five-year outcomes and postoperative complications after total temporomandibular joint replacement with two stock prosthetic systems. Br J Oral Maxillofac Surg. 2020;58(1):69–74.	Confounding
Hussain OT, Sah S, Sidebottom AJ. Prospective comparison study of one-year outcomes for all titanium total temporomandibular joint replacements in patients allergic to metal and cobalt-chromium replacement joints in patients not allergic to metal. Br J Oral Maxillofac Surg. 2014;52(1):34–7.	Confounding
Kanatsios, S. Thomas, A.M. Tocaciu, S. Comparative clinical outcomes between stock vs custom temporomandibular total joint replacement systems. J Craniomaxillofac Surg. 2022;50:322–7.	Confounding
Mehra P, Arya V, Henry C. Temporomandibular joint condylar osteochondroma: complete condylectomy and joint replacement versus low condylectomy and joint preservation. J Oral Maxillofac Surg. 2016;74(5):911–25.	Confounding
Mehra P, Henry CH, Giglou KR. Temporomandibular joint reconstruction in patients with autoimmune/connective tissue disease. J Oral Maxillofac Surg. 2018;76(8):1660–4.	Confounding
Rikhotso RE, Sekhoto MG. Surgical treatment of temporomandibular joint ankylosis: our experience with 36 cases. J Craniofac Surg. 2024;35(6):e536–40.	Confounding
Saeed N, Hensher R, McLeod N, Kent J. Reconstruction of the temporomandibular joint autogenous compared with alloplastic. Br J Oral Maxillofac Surg. 2002;40(4):296–9.	Confounding
Siegmund BJ, Winter K, Meyer-Marcotty P, Rustemeyer J. Reconstruction of the temporomandibular joint: a comparison between prefabricated and customized alloplastic prosthetic total joint systems. Int J Oral Maxillofac Surg. 2019;48(8):1066–71.	Confounding
Vorrasi, J. Harris, H. Karras, M. Basir Barmak, A. Kolokythas, A. Prosthetic temporomandibular joint replacement (TJR): stock or custom? A single institution pilot comparison. Oral Surg Oral Med Oral Pathol Oral Radiol. 2023;135(2)	Confounding
Wolford LM, Dingwerth DJ, Talwar RM, Pitta MC. Comparison of 2 temporomandibular joint total joint prosthesis systems. J Oral Maxillofac Surg. 2003;61(6):685–90; discussion 90.	Confounding
Wolford LM, Amaya P, Kesterke M, Pitombeira Pinto L, Franco P. Can patients with metal hypersensitivity requiring TMJ total joint prostheses be successfully treated with all-titanium alloy mandibular components? J Oral Maxillofac Surg. 2022;80:599−613.	Confounding
Yadav P, Roychoudhury A, Bhutia O. Strategies to reduce re-ankylosis in temporomandibular joint ankylosis patients. Br J Oral Maxillofac Surg. 2021;59:820–5	Confounding
Zou L, Zhao J, He D. Preliminary clinical study of Chinese standard alloplastic temporomandibular joint prosthesis. J Craniomaxillofac Surg. 2019;47(4):602–6.	Confounding

### Risk of bias in studies

**Figure 2:**
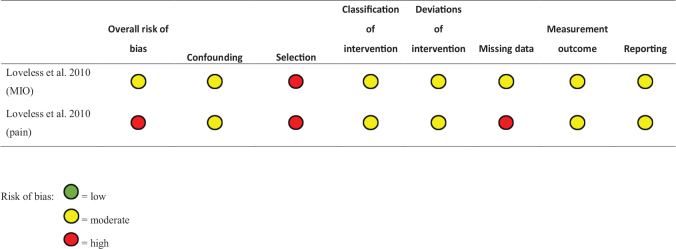
Risk of bias assessment using ROBINS-I-tool.

The included study is presented in Figure 2. The study met the criteria for moderate risk of bias.

### Study characteristics

The included study compared TJR with interpositional arthroplasty (IA) [25]. The study had a retrospective study design and included treatments from two different centers. The reason for treatment was TMJ ankylosis.

The study by Loveless et al. showed increased maximum interincisal opening (MIO) and reduced pain for both groups. The MIO improvement was greater in the IA group; but after regression analysis, the difference was not significant. Pain reduction was greater for TJR, but not significant. For this measure, data was acquired only for 50% of the patients in TJR and 45% of the patients in IA (Table 5).

**Table 5:** Characteristics of included study with moderate risk of bias.

Study variable	TJR	IA
Study population		
Sample size (*n* = 36)	14 (39%)	22 (61%)
Women	11 (79%)	14 (64%)
Etiology		
Iatrogenic	5 (36%)	4 (18%)
Systemic	3 (21%)	4 (18%)
Other	6 (43%)	14 (64%)
Unilateral treatment	5 (36%)	16 (73%)
Age (yr)	45.6 ± 7.3	36.6 ± 14.9
Median follow-up (mo)	12 (0.3–105)	12 (0.3–71.9)
Previous operations	3.7 ± 3.5	1.1 ± 1.2
Outcome		
MIO (mm)		
Preoperative	15.6 ± 7.3	10.3 ± 8.5
Postoperative	24.9 ± 10	28 ± 8.6
Change	9.4 ± 6.7	18 ± 9.7
Regression coeff.^a^	0 (ref.)	7.2 (95% CI: -0.2–14.6)
Pain (VAS)		
Preoperative	6.1 ± 3.6 (*n* = 7)	2.3 ± 3.3 (*n* = 10)
Postoperative	3.1 ± 3.5 (*n* = 7)	2.2 ± 2.9 (*n* = 10)
Change	3 ± 3.1 (*n* = 7)	0.1 ± 1.3 (*n* = 10)
Regression coeff.^b^	0 (ref.)	-1.9 (95%CI: -4.6–0.8)

IA: interpositional arthroplasty; TJR: total joint replacement.

a Adjusted for institution, age, laterality and previous procedures.

b Adjusted for geography and preoperative pain scores.

### Certainty of evidence

The certainty of evidence for MIO and pain reduction was assessed and was found very low, as shown in Table 6.

**Table 6:** Summary of findings.

Change in function, clinical outcomes after TMJ arthroplasty							
**Population:** Patients with a documented diagnosis of bony or fibrous TMJ ankylosis. **Exposure:** TJR, Total Joint Replacement **Comparison:** IA, Interpositional Arthroplasty **Outcome:** Increase in MIO, maximal incisial opening, reduction of pain							
Outcomes		Change in clinical outcomes		Mean difference	Quality of evidence according to GRADE	Downgrading	Interpretation
		TJR	IA				
Improvement in Mio	Loveless et. al	9.4 ± 6.7	18 ± 9.7	7.2 (95% CI: -0.2–14.6)	⊕⊖⊖⊖^a,b^ Very low	^a^Downgraded two level due to risk of bias. ^b^Downgraded one level due to imprecision	Not possible to assess outcome to favor either treatment.
Improvement in pain	Loveless et. al	3 ± 3.1 (*n* = 7)	0.1 ± 1.3 (*n* = 10)	-1.9 (95%CI: -4.6–0.8)	⊕⊖⊖⊖^a,b^ Very low	^a^Downgraded two level due to risk of bias. ^b^Downgraded one level due to imprecision	Not possible to assess outcome to favor either treatment.

**GRADE Working Group grades of evidence**

**High quality:** Further research is very unlikely to change our confidence in the estimate of effect.

**Moderate quality:** Further research is likely to have an important impact on our confidence in the estimate of effect and may change the estimate.

**Low quality:** Further research is very likely to have an impact on our confidence in the estimate of effect and is likely to change the estimate.

**Very low quality:** We are very uncertain about the estimate.

## Discussion

This SR could identify and include only one study that met our criteria.

It was therefore not possible to draw any broader conclusions from the results in this particular study. The included study by Loveless et. al compared TJR with IA in a retrospective cohort from two clinical centers [25]. The study has limitations, such as small sample size and heterogeneous study groups. Most likely, the difference between the groups in that study regarding the pre-existing group differences may explain their results. The authors discussed this matter and acknowledged that the groups were heterogeneous and that the TJR group most likely had a more severe condition and as well was more likely to have had more previous surgeries. Their results must therefore be interpreted with caution. The choice of TJR or IA should not be made from expected differences in clinical outcome.

Many of the studies investigating TJR were observational and did not include control groups. There is a high risk for confounding factors in these type of studies. We could not identify any randomized clinical trials.

This SR reveals a significant gap in the literature: there are no randomized controlled trials on this critical topic, and even observational studies with control groups are scarce. The field of TMJ reconstruction with alloplastic prostheses is rapidly evolving, with new systems continually entering the market [26, 27]. Despite this fact, not even the oldest and most established TJR alloplastic brands have been sufficiently reviewed in a controlled setting. This review highlights the urgent need for further scientific efforts, including well-designed trials capable of demonstrating the comparative effectiveness of alloplastic TJR against other treatment modalities. However, a knowledge gap has been identified, strongly suggesting randomized trials to be conducted, enabling measurements of the true effects of TMJ TJR with alloplastic prosthesis.
